# Hope, anxiety, PTSD and depression in COVID-19-bereaved family members

**DOI:** 10.1192/j.eurpsy.2023.507

**Published:** 2023-07-19

**Authors:** F. Franza, A. Franza, A. De Paola, F. Papa, C. Esposito, B. Solomita

**Affiliations:** ^1^Psychiatry, Psychiatric Rehabilitation Centre Villa dei Pini, Avellino; ^2^Neuroscience, Neamente Association, MERCOGLIANO, Italy

## Abstract

**Introduction:**

Sadness, nostalgia, deep discomfort, guilt and feelings of loss, hopelessness are just some of the emotions that overwhelm people who are experiencing the death of a loved one (Franza *et al*. Psychiatr Danub 2022; 34 (8) 60-63). The unusual mourning process in the time of COVID-19 challenges the usual process of coping with loss. The absence of the funeral rite and coping in time of COVID-19 affects the grieving process. The consequences of “bodiless” bereavement in survivors of people who died during the COVID-19 pandemic may be similar to Post Traumatic Stress Disorder (PTSD) (Spurio. Psychiatr Danub 2021; 33 (Suppl 9) 102-107).

**Objectives:**

To evaluate the effects of the absence of the funeral rite on anxiety, depression, PTSD and hope in family members of people who have died from COVID-19.

**Methods:**

In our observational study, 23 family members (12 females; 11 males; mean age: 48.56 yrs) who experienced a bereavement of a loved one without participation in funeral rites due to COVID-19 restrictions were recruited. They had turned to mental health professionals (psychiatrists and psychologists) as suffering from anxiety and depressive disorders. The subjects interviewed between the months of May 2020 and July 2020 (T0) were administered the following evaluation scales: Beck Hopelessness Scale (BHS), Beck Depression Inventory -2 (BDI-II); Anxiety Zung, and PTSD Checklist for DSM-5 (PCL-5).

The same scales were administered after 1 year (T1) and after two years (T2).

All the relevant data were analysed using EZAnalyze Version 3.0, Microsoft Excel Add-ln. Repeated Measures ANOVA Variables used for analyzing scales scores.

**Results:**

The main results are shown in Table 1. High values of hopelessness, anxiety and depressive symptoms were observed in T0. The score was reduced in the following times. In BHS the ANOVA results indicate that at least two of the repeated measures differed significantly (P – Unadjusted: T0 and T2: .003, T1 and T2: .009; P – Bonferroni: T0 and T1: .009, T1 and T2: 0.28). Similar results were highlighted in the Zung and DBI-II scales. These results indicate high levels of anxiety and depression at the beginning of the observation period (T0). The results for the assessment of PTSD indicate statistically significant differences (P. .000, Eta Squared: .378).

**Image:**

**Figure:**
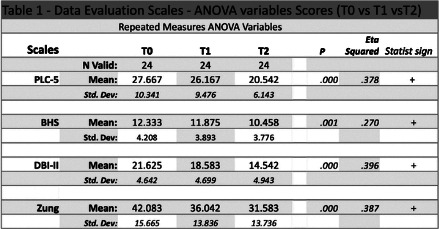

**Conclusions:**

Our little study evaluated some psychological factors in the emotional process of “normal” and complicated mourning. The loss of a loved one is inevitably an extremely painful event and is accompanied by a series of highly emotional experiential pathways. In the first months after death, family members have high levels of anxiety, depression, and hopelessness. There is a need to deepen the study with a higher number of participants and with a comparison with “normal” bereavement

**Disclosure of Interest:**

None Declared

